# Cost-Effectiveness of Avelumab Maintenance Therapy Plus Best Supportive Care vs. Best Supportive Care Alone for Advanced or Metastatic Urothelial Carcinoma

**DOI:** 10.3389/fpubh.2022.837854

**Published:** 2022-04-27

**Authors:** Qian Xie, Hanrui Zheng, Ye Chen, Xingchen Peng

**Affiliations:** ^1^West China Hospital, Sichuan University, Chengdu, China; ^2^West China School of Medicine, West China Hospital, Sichuan University, Chengdu, China

**Keywords:** programmed cell death ligand 1, avelumab, cost-effective, advanced or metastatic urothelial cancer, maintenance therapy

## Abstract

**Objective:**

Avelumab (MSB0010718C) is a fully human anti-programmed cell death ligand 1(PD-L1) antibody against PD-L1 interactions and enhances immune activation against tumor cells in the meantime. Avelumab has been approved for locally advanced or metastatic urothelial cancer (mUC) after disease progression in several countries. We therefore conducted this study to evaluate the cost-effectiveness of avelumab maintenance therapy for advanced or mUC from the perspective of the United States (US) and China payer.

**Methods:**

A Markov simulation model was performed based on clinical trial JAVELIN Bladder 100. Utilities and costs adopted in this analysis were derived from published literature and clinical trials. Incremental cost-effectiveness ratios (ICERs) were calculated to compare the avelumab maintenance therapy group (AVE group) and the best supportive care group (CON group).

**Results:**

The ICER of the AVE group compared with the CON group were $38,369.50 and $16,150.29 per QALYs in the overall population and in the PD-L1–positive population, respectively. While the ICER of AVE group compared with CON group were $241,610.25 and $100,528.29 per QALYs in the overall population and in the PD-L1–positive population, respectively.

**Conclusion:**

Avelumab maintenance therapy was a cost-effective first-line treatment compared with BSC in patients with mUC which were not progressed with platinum-based chemotherapy not only in the PD-L1–positive population but also in the overall population based on the current willingness to pay (WTP) of $150,000 in the US. It was not cost-effective both in the overall population and in the PD-L1 positive population at the WTP threshold of $30,447.09 in China.

## Introduction

It is estimated that about 81,000 new cases of bladder cancer are diagnosed per year and 17,980 deaths in 2020 in the United States (US) (1, 2). Approximately 90% of bladder cancer cases are urothelial carcinoma (UC) with the highest incidence in Europe and North America (3, 4). Nearly 10,000 new cases are diagnosed and more than 3,000 deaths occurred in China annually (5). Patients with advanced or metastatic urothelial cancer (mUC) are usually associated with a poor prognosis, with a 5-year survival rate of less than 5% (6). In patients with mUC, cisplatin-based combination chemotherapies are recommended as standard first-line therapy (7).

In recent years, more attention was drawn to the immune checkpoint inhibitors (ICIs) which generate immune antitumor mechanisms. Programmed cell death 1 (PD-1) and programmed cell death ligand 1(PD-L1) demonstrated a significant benefit on multiple tumor diseases compared with other immunotherapy (8). Patients with mUC currently still have risks for relapse after first-line chemotherapies. Therefore, a maintenance therapy that has both high efficacy and tolerability in these patients is of great importance.

The JAVELIN Bladder 100 trial which was a multiple-center, open-label, phase 3 trial demonstrated that avelumab as first-line maintenance therapy in patients with mUC in disease progressed free stage with platinum-based chemotherapy resulted in significantly benefit both in progressed-free survival (PFS) and overall survival (OS) than best supportive care (BSC) alone (9). Avelumab (MSB0010718C) is a fully human anti–PD-L1 antibody against PD-1/ PD-L1 interactions and enhances immune activation against tumor cells in the meantime. Avelumab has been approved for locally advanced or mUC after disease progression in several countries (10, 11).

Maintenance therapy with new treatment agent after first-line chemotherapy has shown significant efficacy in several tumor types (12). However, treatments with ICIs were usually expensive, so reasonable value is a great concern both for healthcare payers and patients. Healthcare budgets are undergoing increasing pressure worldwide, mainly due to increased costs associated with newly-developed treatment innovations. Due to the willingness-to-pay (WTP) threshold and cost estimates are usually region-specific, so we conducted this study to evaluate the cost-effectiveness of avelumab maintenance therapy plus BSC vs. BSC alone for advanced or mUC from the payer perspective both in the US and China.

## Methods

### Patients and Treatment

Our analysis was based on the multiple-center, open-label, phase 3 clinical trial (JAVELIN Bladder 100). According to the JAVELIN Bladder 100 Clinical Trial, patients with histologically confirmed, locally advanced, or metastatic urothelial carcinoma who had finished four to six cycles of platin-based chemotherapy with a treatment-free interval of 4 to 10 weeks were randomly assigned to the two groups in a 1:1 ratio. Avelumab was given intravenously at a dose of 10 mg per kg of body weight every 2 weeks plus BSC (AVE group, 350 patients) or BSC alone (CON group, 350 patients). The median duration of treatment was 24.9 weeks in the AVE group and 13.1 weeks in the CON group. Imaging tests were performed every 8 weeks. Adverse events were graded according to the National Cancer Institute Common Terminology Criteria for Adverse Events, version 4.03 (9).

A total of 700 patients from 197 sites in 29 countries were enrolled; the characteristics at baseline, including age, baseline metastasis before chemotherapy and PD-L1–positive proportion. were well-balanced between the two treatment groups. In the overall population, the OS was 21.4 months (95% CI, 18.9 to 26.1) and 14.3 months (95% CI, 12.9 to 17.9) in the AVE group and CON group, respectively. The median PFS was 3.7 months (95% CI, 3.5 to 5.5) and 2 months (95% CI, 1.9 to 2.7) in the AVE group and CON group, respectively. While in the PD-L1–positive population, OS and PFS were significantly longer in the AVE group than in the CON group (9).

### Model Structure

**Figure 1:**
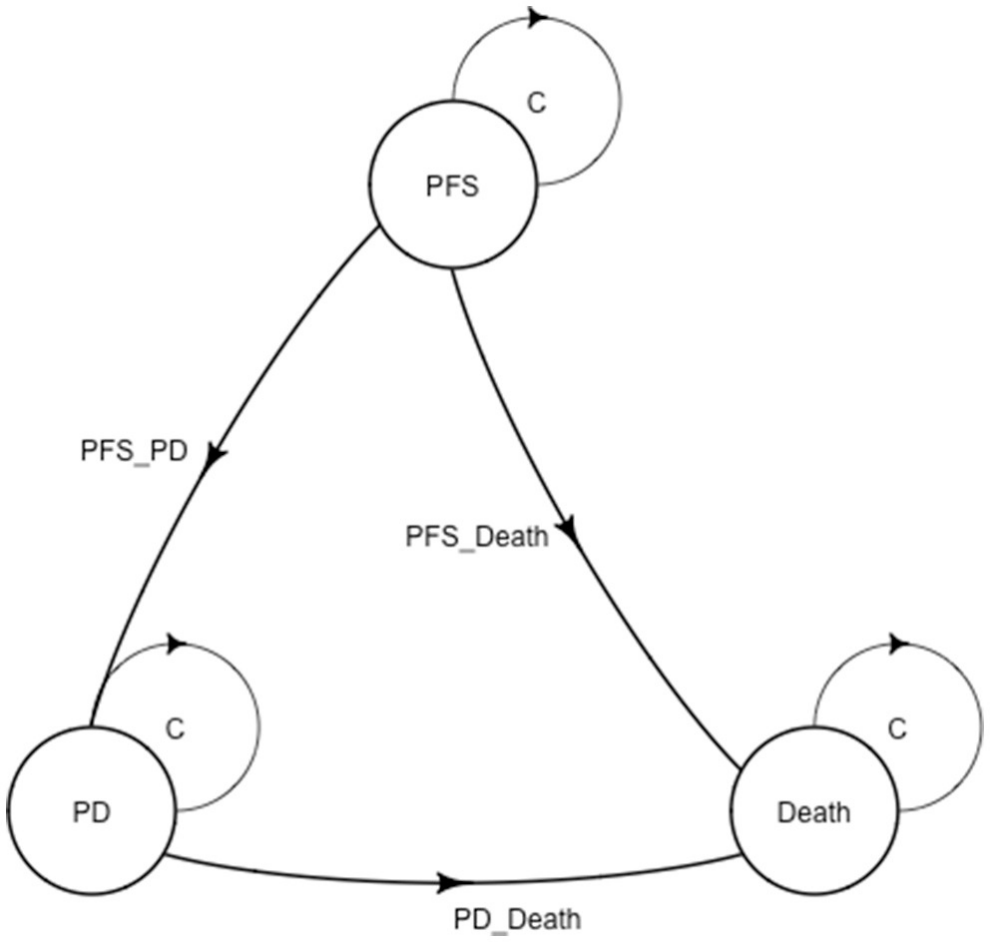
A Markov structure was built to perform the analysis. PFS, progression-free survival; PD, progressive disease.

**Figure 2:**
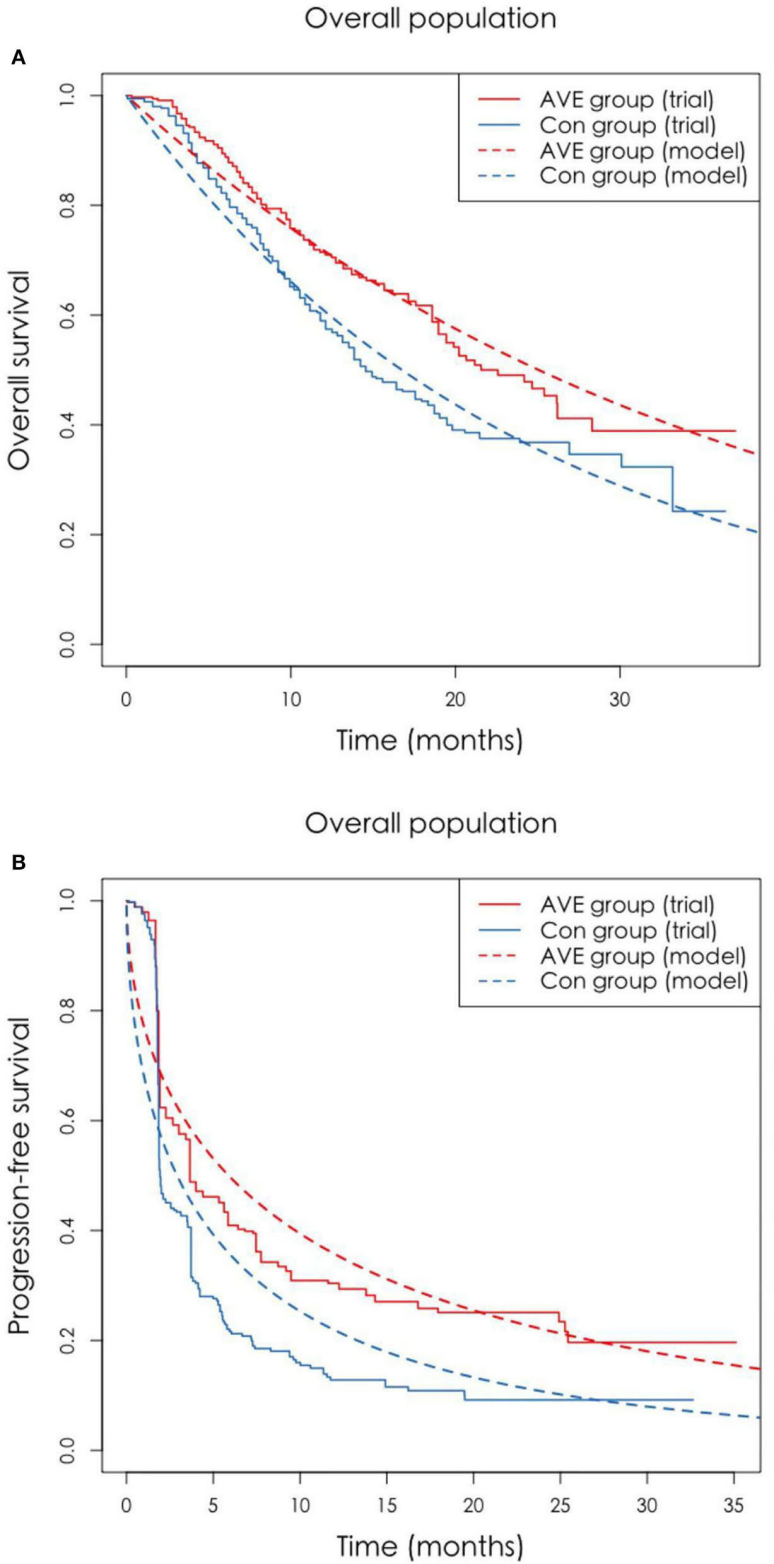
**(A,B)** The original Kaplan-Meier PFS and OS curves from the trial and fitted curves (Weibull distributions). OS, overall survival; PFS, progression-free survival.

The Markov model was conducted using TreeAge Pro 2020 software (TreeAge, Williamstown, Massachusetts). Statistical software package R (Version 4.0.5; R Core Team, 2021) was used for data description, model building, and sensitivity analysis. For model building, three health states: progression-free survival (PFS), progressive disease (PD), and death with time varied transition probability were considered in a Markov model. All the patients entered in the PFS state at first. And then patients could either progress to PD state or death, while patients in the PD state could either progress to death or still be in PD state. Patients may also die in either PFS state or PD state (Figure 1). Firstly, the time varied transition probabilities were estimated using published OS and PFS survival curves in the JAVELIN Bladder 100 Clinical Trial. Function getpoints in package IPDfromKM were used to extract original survival time and corresponding survival probabilities; function getIPD was used to construct individual patient data (IPD), and a parametric Weibull model was built based on the IPD (Figure 2). Then the time varied transition probability from PFS to PD and PD to death in each cycle were estimated using the function flexsurvreg in heemod based on the formula: P(t → St+1)=1–exp[λ(t)γ – λ(t+1)γ)]. Transition probability from PFS to death for each age group was estimated based on USA life tables in the model. The model cycle used for the base case was 1 month and a time horizon of 10 years. To improve estimates accuracy of survival time, pseudo-individual patient data were generated using the algorithm based on published literature (13).

### Costs and Utilities

Drug acquisition costs were calculated based on the average sale price from Drugs.com 2021 (14). We also calculated costs of BSC, Adverse Events (AEs)-related treatments, pretreatment, PD-L1 status tests, routine imaging tests, and subsequent treatment based on published literatures (15–19). As avelumab has not been approved by National Medical Products Administration (NMPA) in mainland China, we refer to the price in Hong Kong, China. All costs were measured in US dollars (USD) based on the exchange rate on Feb 8, 2022 (1 USD = 6.36 CHY). The utility of two groups was estimated by dividing the Quality of Life Questionnaire Core 30 items (QLQ-C30) score by 126. One stands for perfect health and 0 stands for death. Health state utilities were calculated according to the previously published literature (20). The same utilities were applied in both the AVE and CON groups. The costs and utilities are presented in Table 1.

**Table 1:** Input parameters for cost-effectiveness analysis.

**Parameters**	**US value(range)**	**China value(range)**	**Distribution**
**Utilities**			
PFS^a^	0.84 (0.68–1.00)	0.84 (0.68–1.00)	beta
PD^b^ **Cost**^c^	0.80 (0.64–0.96)	0.80 (0.64–0.96)	beta
Cost of PD-L1 test (21)	431.28 (345.03–517.54)	157.23 (125.78–188.68)	gamma
Cost of pretreatment (15)	6.96 (5.57–8.35)	1.57 (1.26–1.88)	gamma
Cost of AE^d^ in CON group (16–19)	74.89 (59.91–89.87)	29.73 (23.78–35.68)	gamma
Cost of AE^d^ in AVE group (16–19) Cost of imaging examination (21) Cost of PD^b^ (21) **In whole population**	111.68 (89.34–134.02) 1,137.67 (910.14–1,365.20) 2,396.70 (1,917.36–2,876.04)	51.33 (41.06–61.60) 163.96 (131.17–196.75) 376.83 (301.46–452.20)	gamma gamma gamma
Cost of avelumab (14) Cost of BSC^e^ in AVE group (15) Cost of BSC^e^ in CON group (15) **In PD1 positive population**	22,022.59 (17,618.07–26,427.11) 4,324.24 (3,387.39–5,081.09) 4,113.4 (3,290.72–4,936.08)	172,637.26 (138,109.81–207,164.71) 2,726.00 (2,180.80–3,271.20) 2,642.93 (2,114.34–3,171.52)	gamma gamma gamma
Cost of avelumab (14) Cost of BSC^e^ in AVE group (15) Cost of BSC^e^ in CON group (15) Discount rate, % (22, 23)	14,295.37 (11,436.30–17,154.44) 2,754.38 (2,203.50–3,305.26) 3,917.52 (3,134.02–4,701.02) 3	112,062.00 (89,649.60–134,474.40) 2,754.38 (2,203.50–3,305.26) 3,917.52 (3,134.02–4,701.02) 5	gamma gamma gamma

a *progression-free survival*;

b *progressed disease*;

c *cost per patient per month*;

d *adverse events*;

e *best supportive care*.

The total costs and Quality-Adjusted Life Years (QALYs) were the output in this model. And then we used the incremental costs and QALYs to calculate the incremental cost-effectiveness ratio (ICER; incremental cost per QALY gained). The AVE group will become cost-effective only under the condition that the ICER between the two groups was below the WTP threshold. An annual discount rate of 3 and 5% were adopted to determine the present value of costs and health utilities in US and China, respectively (22, 23).

### Sensitivity Analysis

In the sensitivity analysis part, deterministic sensitivity analysis (DSA), and probabilistic sensitivity analysis (PSA) were considered. For the DSA, a one-way sensitivity analysis was conducted to examine the impact of utility and cost parameters on the ICER. The result of DSA was represented as a tornado plot with the more sensitive parameters in a broader rectangle on the top. For the PSA, a Monte Carlo simulation was run with 1,000 simulations with 1,000 individuals, beta distribution for utility parameters and gamma distribution for cost parameters were considered, respectively. The result of PSA was represented as a cost-effectiveness acceptability curve. The WTP threshold in the US and China were set as $150,000 and $30,447.09 per QALY, respectively, based on the published literature (24, 25).

## Results

### Base-Case Results

Our base-case analysis showed that over a 10-year life horizon, AVE group gained.21 QALYs when spending $1,917.13, while CON group gained.17 QALYs when spending $436.35 in the overall population, which means patients in the AVE group gained.04 QALYs more than patients in the CON group. While in the PD-L1–positive population, the AVE group gained.07 QALYs more than patients in the CON group when spending $1,130.52 extra. The ICER of AVE group compared with CON group were $38,369.50 and $16,150.29 per QALYs in the overall population and in the PD-L1–positive population, respectively, both below the WTP threshold of $150,000 in the US. While the ICER of AVE group compared with CON group were $241,610.25 and $100,528.29 per QALYs in the overall population and in the PD-L1–positive population, respectively, both exceed the WTP threshold of $30,447.09 in China (Table 2).

**Table 2:** The summary of results of the cost-effectiveness analysis.

**Parameters**	**AVE group**	**CON group**	**Increment**
**Unite States**			
**Overall population**			
Cost,USD	1,971.13	436.35	1,534.78
QALYs^f^	0.21	0.17	0.04
ICER^g^	-	-	38,369.50
**PD-L1+** **population**			
Cost,USD	1,597.66	467.14	1,130.52
QALYs^f^	0.25	0.18	0.07
ICER^g^	-	-	16,150.29
**China**			
**Overall population**			
Cost,USD	9,794.48	130.07	9,664.41
QALYs^f^	0.21	0.17	0.04
ICER^g^	-	-	241,610.25
**PD-L1+** **population**			
Cost,USD	7,171.91	134.93	7,036.98
QALYs^f^	0.25	0.18	0.07
ICER^g^	-	-	100,528.29

f *QALYs, quality-adjusted life year; ^g^ICER, Incremental cost-effectiveness ratio*.

### Sensitivity Analysis

**Figure 3:**
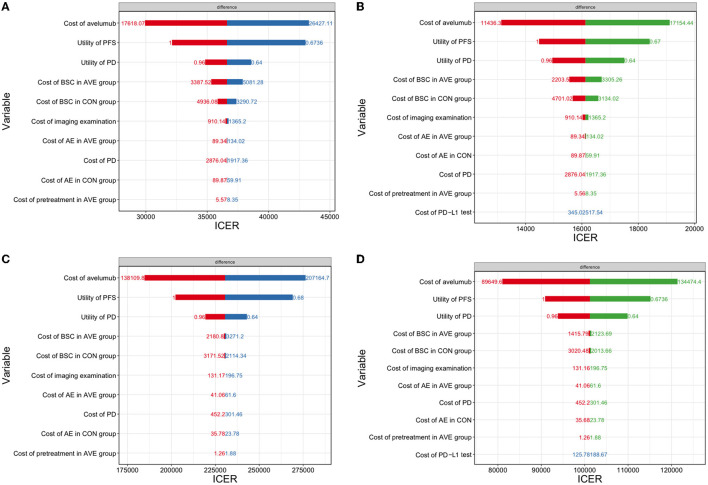
A one-way sensitivity analysis was presented in the tornado diagram. The impact of different parameters on the ICER was listed. **(A)** The overall population in the US; **(B)** The PD-L1–positive population in the US; **(C)** The overall population in China; **(D)** the PD-L1–positive population in China. ICER, incremental cost-effectiveness ratio; PFS, progression-free survival; PD, progressive disease.

One-way sensitivity analyses were shown in Figure 3, which demonstrated that the cost of avelumab and utility of PFS were the most sensitive influential factors in all of the four scenarios analysis. Other variables, such as the cost of PD-L1 test, cost of BSC, and cost of PD, had a minor influence on the results.

**Figure 4:**
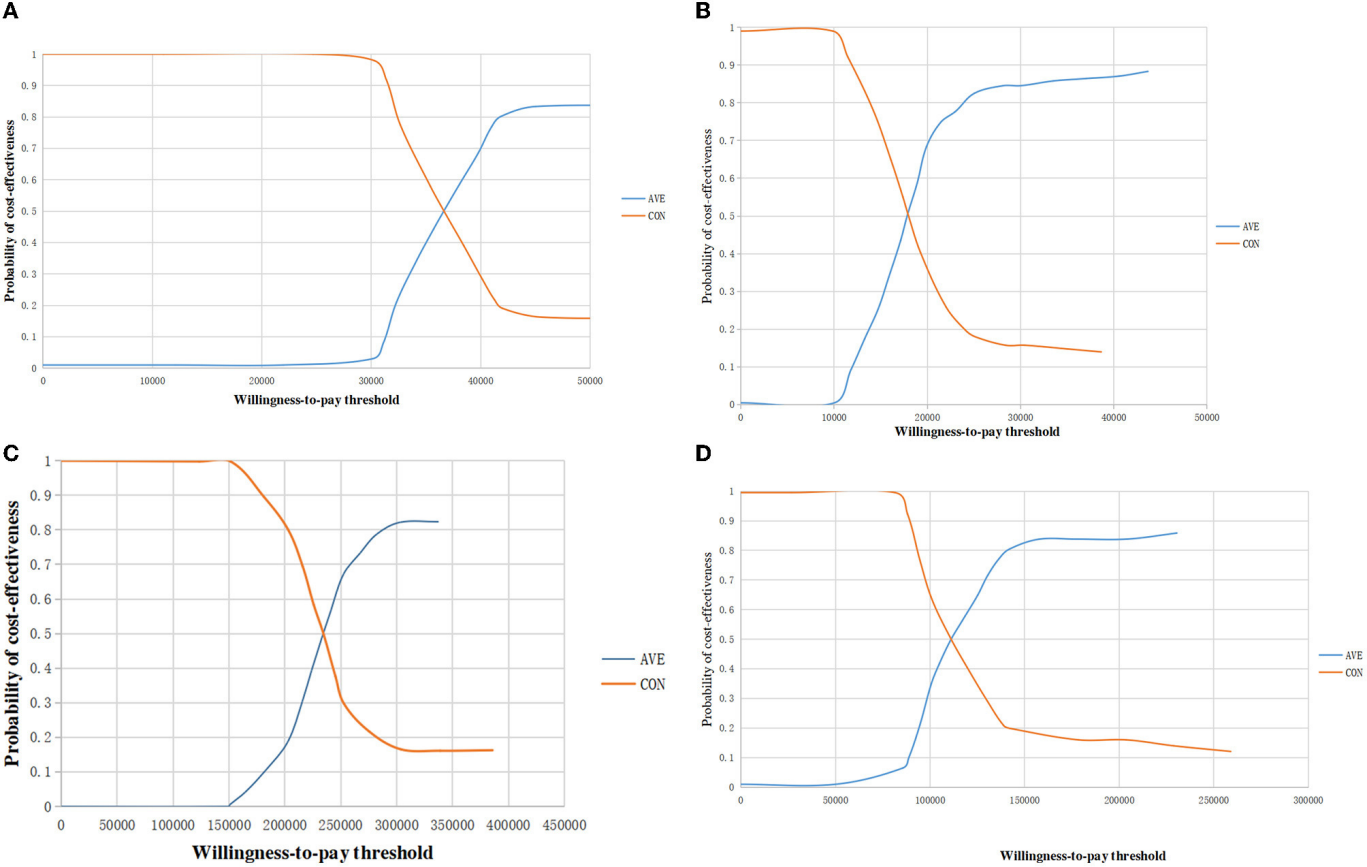
The acceptability curve of cost-effectiveness showed the probability at current WTP threshold. **(A)** The overall population in the US; **(B)** The PD-L1–positive population in the US; **(C)** The overall population in China; **(D)** The PD-L1–positive population in China. WTP, willingness to pay; QALYs, quality-adjusted life year.

The acceptability curve in Figure 4 displays the results of the probabilistic sensitivity analysis, which indicated that, at current WTP in the two countries, avelumab maintenance therapy was considered cost-effective both in the overall population and in the PD-L1 positive population in the US, however it was not cost-effective both in the overall population and in the PD-L1 positive population in China.

## Discussion

The five-year OS rate for mUC is poor and recently a few studies in the US reported that only a small proportion of patients with mUC accepted subsequent lines therapy after first-line therapy, almost half of the patients did not accept treatments for advanced or metastatic disease (26). Therefore, maintenance treatment is particularly important which can reduce the recurrence rates for patients with mUC. Most notably, the US is a developed country with a high-income and well-developed health system, while the proportion of underutilization of therapy may be even lower among patients in lower-income countries. So, we conducted the cost-effectiveness analysis both in the developed and developing countries.

The clinical trial demonstrated that avelumab maintenance therapy in mUC gained significant longer PFS and OS and this approach could become part of clinical practice in the future (27). Avelumab maintenance therapy improves life expectancy compared to the control group; however, evaluation of cost-effectiveness always depends both on the costs and efficacy. The JAVELIN Bladder 100 Clinical Trial demonstrate that the OS benefit with AVE group was greater compared with the CON group in the PD-L1–positive population than in the overall population, which makes the AVE group even more cost-effective in the PD-L1–positive population. The AVE group provides QALY gains at ICERs that are lower than the WTP of $150,000 per QALY in the US in both populations. Then it is worth our consideration is that whether a PD-L1 test is necessary before avelumab maintenance therapy in patients with mUC in the US because avelumab maintenance therapy was cost-effective both in the PD-L1–positive population and in the overall population. While in some lower-income countries, a PD-L1 test may be necessary to maximize outcomes and minimize costs due to a lower WTP and shortage of health resources.

Our study demonstrated that the avelumab maintenance therapy was not cost-effective in both populations in China, mainly because of the extremely high price of avelumab. However, our analysis was based on the price of avelumab in Hong Kong, after the approval of avelumab in mainland China, the price has a great chance to be lower than that in Hong Kong now, which may lead to overestimate the cost of China in our analysis. So, we calculated that the price of avelumab should be 86.24 and 82.17% lower than the current price, avelumab maintenance therapy will be cost-effective in the overall population and PD-L1–positive population, respectively, at the current WTP of China. Perhaps this will send some information to the Chinese health payer for considering appropriate resource allocation.

A recent study based on the JAVELIN Bladder 100 Clinical Trial demonstrate that avelumab maintenance therapy was cost-effective both in the overall population and PD-L1–positive population in the US, which was similar to our results (28). Pembrolizumab, another ICIs, was demonstrated as a cost-effective first-line treatment for patients with mUC whose tumors strongly expressed PD-L1 in Sweden and the US at the WTP threshold of £100,000 and $150,000 per QALY, respectively (20, 21). Although pembrolizumab shows great efficacy for patients with UC, recently the National Institute for Health and Care Excellence (NICE) decided to reject coverage of pembrolizumab for platinum-refractory UC due to its failure to meet their threshold of £50,000/QALY in the UK, which was also a good illustration for the growing importance of cost-effectiveness analysis in expensive treatments (29).

Our study still has limitations as follows. First, we extracted clinical data from published clinical trials instead of real-world patients, which may not totally reflect situations. In the PD-L1–positive population, OS in the AVE group was not reached in the published JAVELIN Bladder 100 trial. So, we simulated survival curves based on the trial which were good fit. Second, because quality-of-life data were not available from the JAVELIN Bladder 100 trial, the utilities were obtained from published literature which were the analysis of ICIs on urothelial carcinoma. Third, AEs-related treatments in China used in the analysis were based on Chinese expert opinion, which may cause bias. Fourth, we obtained the cost of PD from published literatures and applied the same costs in the two groups, which may differ from real-world treatment choices, because the second line therapy choice can be another ICIs or chemotherapy, prices of which can vary widely, however according to the sensitivity analyses, cost of PD had a minor influence on the results.

## Conclusions

Our study demonstrated that avelumab maintenance therapy was a cost-effective first-line treatment compared with BSC in patients with mUC which were not progressed with platinum-based chemotherapy not only in the PD-L1–positive population but also in the overall population based on the current WTP of $150,000 in the US. It was not cost-effective both in the overall population and in the PD-L1 positive population at the WTP threshold of $30,447.09 in China. When the price of avelumab is 86.24 and 82.17% lower than the current price, avelumab maintenance therapy will be cost-effective in the overall population and PD-L1–positive population, respectively, at the current WTP of China.

## Data Availability Statement

The original contributions presented in the study are included in the article/supplementary material, further inquiries can be directed to the corresponding author/s.

## Author Contributions

XP and QX: conception and design. QX and HZ: analysis and interpretation of data. XP, QX, and YC: development of methodology. All authors contributed to the article and approved the submitted version.

## Funding

The work was supported by the Sichuan Province Science and Technology Support Program (No. 2021YFSY008) and the technology Innovation Project of Chengdu Science and Technology Bureau (No. 2019-YF05-00459-SN).

## Conflict of Interest

The authors declare that the research was conducted in the absence of any commercial or financial relationships that could be construed as a potential conflict of interest.

## Publisher's Note

All claims expressed in this article are solely those of the authors and do not necessarily represent those of their affiliated organizations, or those of the publisher, the editors and the reviewers. Any product that may be evaluated in this article, or claim that may be made by its manufacturer, is not guaranteed or endorsed by the publisher.
