# Corrigendum: Cost-effectiveness of avelumab maintenance therapy plus best supportive care vs. best supportive care alone for advanced or metastatic urothelial carcinoma

**DOI:** 10.3389/fpubh.2022.965798

**Published:** 2022-07-26

**Authors:** Qian Xie, Hanrui Zheng, Ye Chen, Xingchen Peng

**Affiliations:** ^1^West China Hospital, Sichuan University, Chengdu, China; ^2^West China School of Medicine, West China Hospital, Sichuan University, Chengdu, China

**Keywords:** programmed cell death ligand 1, avelumab, cost-effective, advanced or metastatic urothelial cancer, maintenance therapy

In the published article, there was an error in Figure 2 where parts 2A and 2C were duplicated in parts 2B and 2D. The caption should also read as “The original Kaplan-Meier PFS and OS curves from the trial and fitted curves (Weibull distributions). OS, overall survival; PFS, progression-free survival.” The corrected figure appears below.

**Figure 2:**
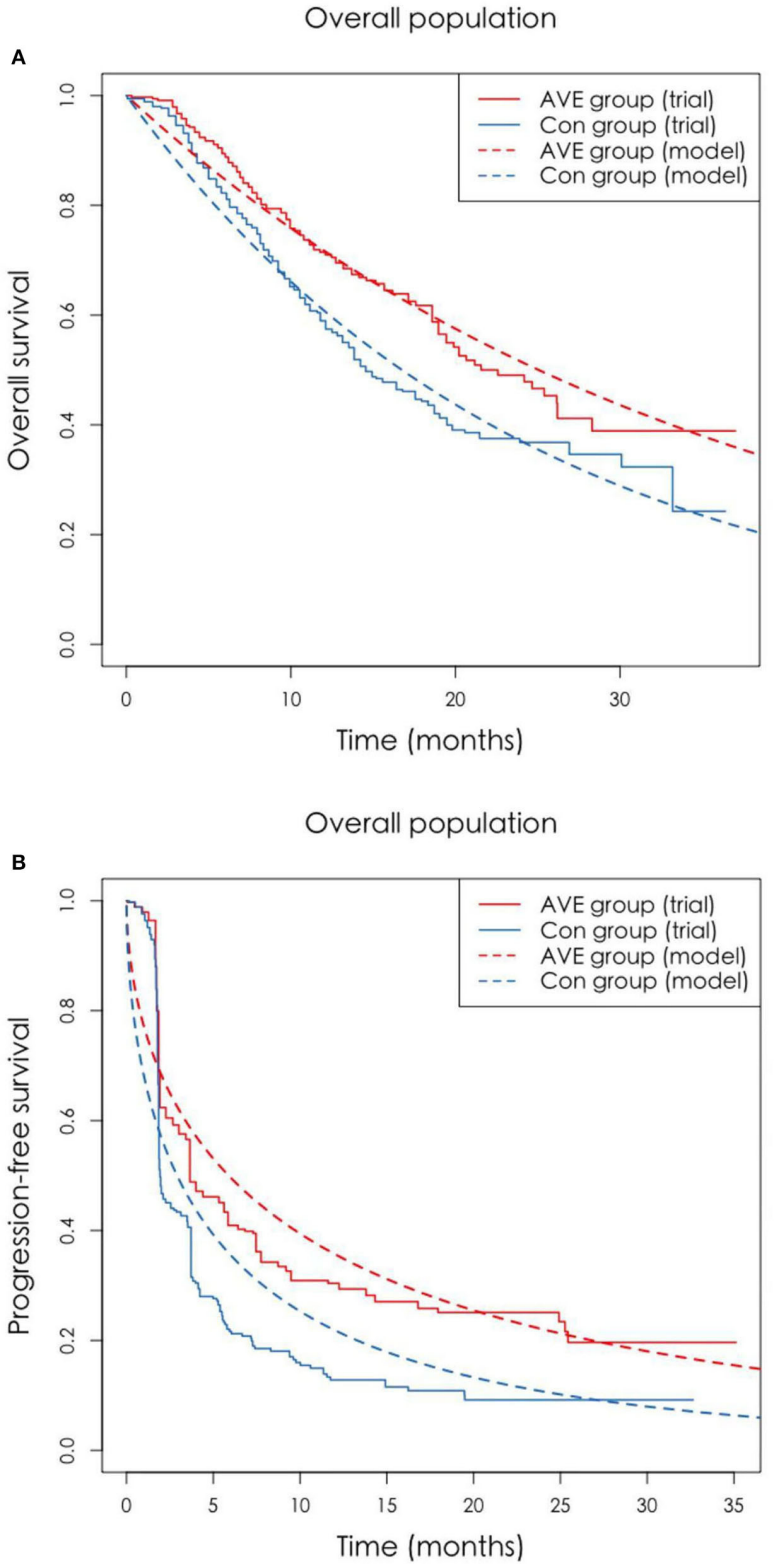
**(A,B)** The original Kaplan-Meier PFS and OS curves from the trial and fitted curves (Weibull distributions). OS, overall survival; PFS, progression-free survival.

The authors apologize for this error and state that this does not change the scientific conclusions of the article in any way. The original article has been updated.

## Publisher's note

All claims expressed in this article are solely those of the authors and do not necessarily represent those of their affiliated organizations, or those of the publisher, the editors and the reviewers. Any product that may be evaluated in this article, or claim that may be made by its manufacturer, is not guaranteed or endorsed by the publisher.

